# Elucidating ecological complexity: Unsupervised learning determines global marine eco-provinces

**DOI:** 10.1126/sciadv.aay4740

**Published:** 2020-05-29

**Authors:** Maike Sonnewald, Stephanie Dutkiewicz, Christopher Hill, Gael Forget

**Affiliations:** 1 Department of Earth, Atmospheric and Planetary Sciences, Massachusetts Institute of Technology, Cambridge, MA 02139, USA.; 2 Department of Earth and Planetary Sciences, Harvard University, Cambridge, MA 02138, USA.

## Abstract

An unsupervised learning method is presented for determining global marine ecological provinces (eco-provinces) from plankton community structure and nutrient flux data. The systematic aggregated eco-province (SAGE) method identifies eco-provinces within a highly nonlinear ecosystem model. To accommodate the non-Gaussian covariance of the data, SAGE uses t-stochastic neighbor embedding (t-SNE) to reduce dimensionality. Over a hundred eco-provinces are identified with the density-based spatial clustering of applications with noise (DBSCAN) algorithm. Using a connectivity graph with ecological dissimilarity as the distance metric, robust aggregated eco-provinces (AEPs) are objectively defined by nesting the eco-provinces. Using the AEPs, the control of nutrient supply rates on community structure is explored. Eco-provinces and AEPs are unique and aid model interpretation. They could facilitate model intercomparison and potentially improve understanding and monitoring of marine ecosystems.

## Teaser

Global marine eco-provinces are determined toward tackling complexity and to explore community structure using unsupervised ML.

## INTRODUCTION

Provinces are regions in the ocean or on land where the complex biogeography has been organized into coherent and meaningful regions (*1*). Such provinces are important for comparing and contrasting locations, characterizing observations, monitoring, and conservation efforts. The intractably complicated and nonlinear interactions that create these provinces make unsupervised machine learning (ML) methods well suited to objectively determine provinces because the covariances within the data manifest as intricate and non-Gaussian. Here, an ML method is presented, which systematically identifies unique marine ecological provinces (eco-provinces) from the Darwin global three-dimensional (3D) physical/ecosystem model (*2*). The term “unique” is used to signify that the identified region is sufficiently different from other regions that they do not overlap. The method is called the systematic aggregated eco-province (SAGE) method. For useful classification, an algorithmic method needs to allow for both (i) global classification and (ii) a multiscale analysis that can be both spatially and temporally nested/aggregated (*3*). In this study, the SAGE method is first presented, and the identified eco-provinces are discussed. The eco-provinces could facilitate understanding of factors controlling community structure, provide insight useful for monitoring strategies, and assist in the tracking of ecosystem changes.

Terrestrial provinces are often classified according to similarity in climate (precipitation and temperature), soil, vegetation, and fauna and used to aid management, biodiversity studies, and disease control (*1*, *4*). Ocean provinces are more difficult to define. The majority of organisms are microscopic, and the boundaries are fluid. Longhurst *et al.* (*5*) provided one of the first global classifications of marine provinces based on environmental conditions. These “Longhurst” provinces were defined using variables such as mixing rates, stratification, and irradiance, along with Longhurst’s extensive experience as a seagoing oceanographer of other key conditions important to the marine ecosystem. The Longhurst provinces have been widely used, for example, to assess primary production and carbon fluxes, to aid fisheries, and to plan in situ observational campaigns (*5*–*9*). Toward defining provinces more objectively, methods such as fuzzy logic and regional unsupervised clustering/statistics have been used (*9*–*14*). Such methods have the goal of identifying meaningful structures that can identify provinces in available observational data. For instance, dynamic seascape provinces (*12*) use self-organizing maps to reduce noise and hierarchical (tree-based) clustering to identify provinces on the basis of regional satellite–derived ocean color products [chlorophyll a (Chl-a), normalized fluorescence line height, and colored dissolved organic material] and physical fields (sea surface temperature and salinity, absolute dynamic topography, and sea ice).

Plankton community structure is of interest as their ecology has a large impact on higher trophic levels and also on carbon uptake and hence climate. Despite this, identifying global eco-provinces based on plankton community structure remains a challenging and elusive goal. Ocean color satellites can potentially offer insight in terms of coarse-grained size fractionation of phytoplankton or suggest dominance of functional groups (*15*), but cannot currently provide details of community structure. More recent surveys [e.g., Tara ocean (*16*)] are providing unprecedented measurements of community structure; there are, at present, only sparse in situ observations at a global scale (*17*). Previous studies have largely determined “biogeochemical provinces” based on identifying biochemical similarities such as in primary production, Chl, and available light (*12*, *14*, *18*). Here, numerical model output [Darwin (*2*)] is used, and eco-provinces are determined in terms of community structure and nutrient fluxes. The numerical model used in this study has global coverage and compares favorably to available in situ data (*17*) and remotely sensed fields (note S1). The numerical model data used in this study have the advantage of global coverage. The model ecosystem consists of 35 phytoplankton and 16 zooplankton types (see Materials and Methods). The model plankton types interact nonlinearly with non-Gaussian covariance structure such that simple diagnostics are not well suited to identifying unique and coherent patterns in the emergent community structure. The SAGE method presented here provides a novel method to examine the complex Darwin model output.

The transformative power of data science/ML techniques can allow overwhelmingly complicated model solutions to reveal complex, but robust, structures in the covariance of data. A robust method is defined as one that can faithfully reproduce results within a given error margin. Determining robust patterns and signals is a challenge even in simple systems. Emergent complexity can appear complicated/intractable until the underlying principles giving rise to the observed patterns are determined. Key processes setting ecosystem composition are inherently nonlinear. The presence of nonlinear interactions can confound robust classification, and methods that make strong assumptions about the underlying statistical distributions of the covariance of data need to be avoided. High-dimensional and nonlinear data are common in oceanography and likely have covariance structures with intricate, non-Gaussian, topology. While data with a non-Gaussian covariance structure can hamper robust classification, the SAGE method is novel as it was designed to allow identification of clusters with arbitrary topology.

**Fig. 1:**
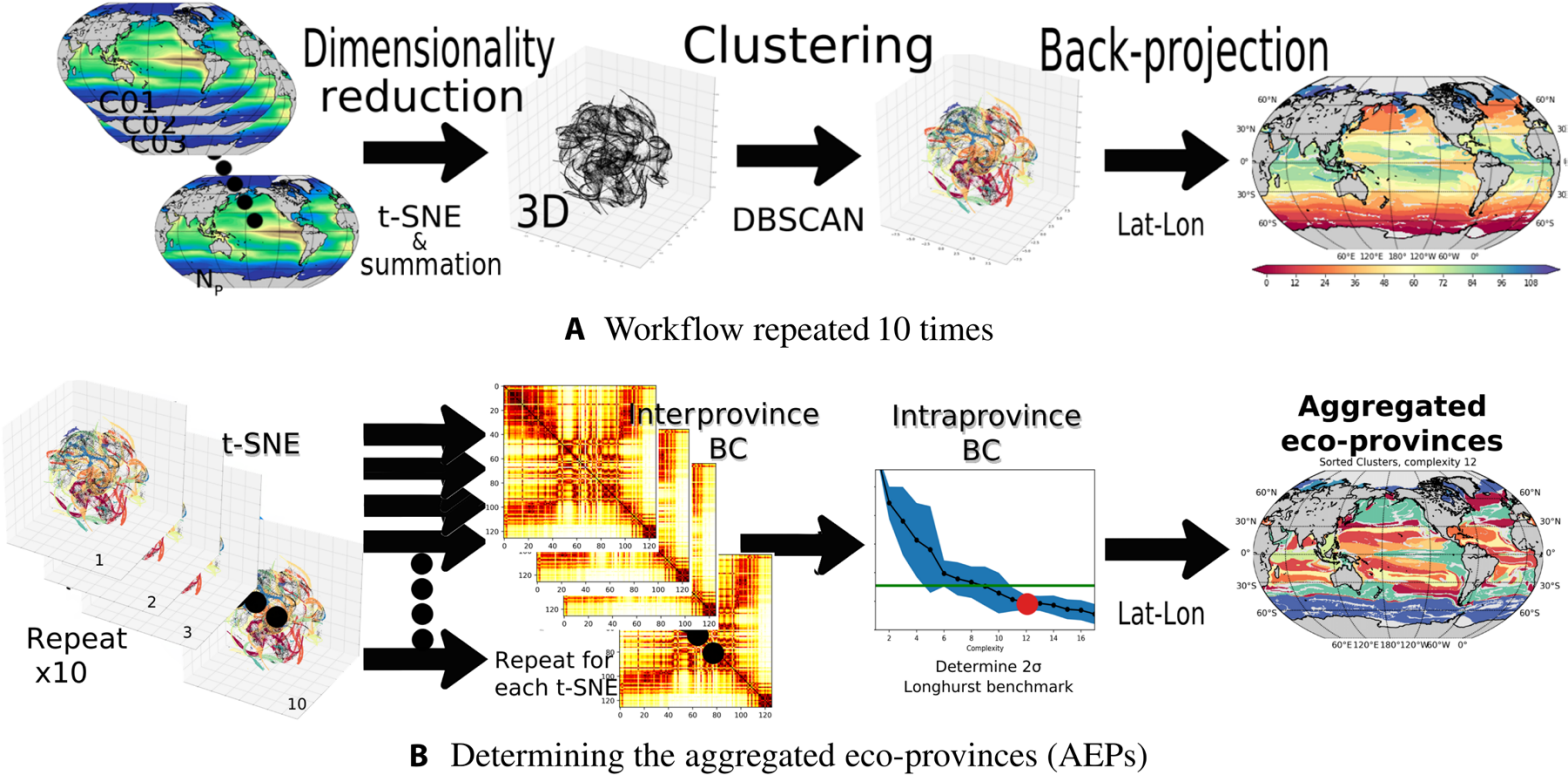
The SAGE method workflow. (**A**) Sketch of the workflow to determine the eco-provinces; raw 55-dimensional data reduced using summation within functional groups to 11-dimensional model output, including biomass of seven functional/trophic groups of plankton and four nutrient supply rates. Negligible values and persistent ice cover areas are discarded. Data are normalized and standardized. The 11-dimensional data are given to the t-SNE algorithm to highlight statistically similar feature combinations. DBSCAN selects the clusters carefully setting parameter values. The data are finally projected back onto a latitude/longitude projection. Note that this process is repeated 10 times, as a slight stochastic element is possible through the application of t-SNE. (**B**) illustrates how the AEPs are arrived at by repeating the workflow in (A) 10 times. For each of the 10 realizations, the interprovince Bray-Curtis (BC) dissimilarity matrix is determined based on the biomass of the 51 phytoplankton types. The BC dissimilarity within the aggregated provinces is determined going from a complexity of 1 AEP to full complexity of 115. The BC benchmark is set by Longhurst provinces.

The goal of the SAGE method is to objectively identify emergent patterns that could help further ecological understanding. Following a clustering-based workflow similar to that in (*19*), the ecological and nutrient flux variables are used to determine unique clusters within the data, referred to as eco-provinces. The SAGE method presented in this study (Fig. 1) first reduces the dimensionality from 55 to 11 dimensions by summing over the a priori defined plankton functional groups (see Materials and Methods). The dimensionality is further reduced by a probabilistic projection onto a 3D space using the t-stochastic neighbor embedding (t-SNE) method. Unsupervised clustering identifies regions of close ecological proximity [density-based spatial clustering of applications with noise (DBSCAN)]. Both t-SNE and DBSCAN are suitable for the inherently nonlinear ecosystem numerical model data. The resulting eco-provinces are then back-projected onto the globe. Over a hundred unique eco-provinces are determined, suitable for regional studies. To consider global coherent ecosystem patterns, the eco-province utility is increased using the SAGE methods ability to aggregate the eco-provinces into aggregated eco-provinces (AEPs). The level of aggregation, termed “complexity,” can be adjusted to a desired level of detail. The minimum complexity number for robust AEPs is determined. The chosen focus is on the SAGE method and on exploring the minimal complexity AEP case to determine controls on the emergent community structures. Patterns can subsequently be analyzed, offering ecological insight. The approach presented here can also be used more widely, for example, for model intercomparison by assessing where similar eco-provinces are found in different models to highlight discrepancies and similarities.

## RESULTS

### Identifying and AEPs

The SAGE method defines eco-provinces using output from a global 3D physical/ecosystem numerical model [Darwin (*2*); see Materials and Methods and note S1]. The ecosystem component consists of 35 phytoplankton types and 16 zooplankton types, with seven a priori defined functional groups: prokaryotes and eukaryotes adapted to low nutrient environments, coccolithophores with calcium carbonate coverings, nitrogen-fixing diazotrophs (often a key missing nutrient), diatoms with siliceous coverings, mixotrophic dinoflagellates that both photosynthesize and graze other plankton, and zooplankton grazers. Sizes span 0.6 to 2500 μm equivalent spherical diameter. The model distribution of size and functional grouping of phytoplankton capture gross features seen in satellite and in situ observations (see figs. S1 to S3). The similarity between the numerical model and the observed ocean suggests that provinces defined from the model may have application to the in situ ocean. Note the caveats that the model only captures some of the diversity of phytoplankton and only some of the range of physical and chemical forcings of the in situ ocean. The SAGE method could lead to a better understanding of the highly regional controlling mechanisms of the model community structure.

The dimensionality of the data is initially reduced by including only the surface, 20-year time-mean sum of biomass, within each plankton functional group. Surface source terms for the flux of nutrients (nitrogen, iron, phosphate, and silicic acid supply) are also included, following earlier studies showing their key roles in setting community structure [e.g. (*20*, *21*)]. Summation over functional groups reduces the problem from 55 (51 plankton and 4 nutrient fluxes) to 11 dimensions. In this initial study, depth and temporal variability are not considered because of computational limitations imposed by the algorithms.

### Dimensionality reduction with t-SNE

The SAGE method is able to identify important relationships between the nonlinear processes and interacting key features in the biomass of functional groups and nutrient fluxes. Obtaining robust, reproducible, provinces is not possible with the 11-dimensional data using learning methods based on Euclidean distances such as *K*-means (*19*, *22*). This is because the underlying distributions of the covariance of key features that define the eco-provinces are not seen to inhabit shapes that are Gaussian. *K*-means, using Voronoi cells (straight lines), is not able to preserve non-Gaussian underlying distribution.

The seven plankton functional group biomasses and the four nutrient fluxes form an 11-dimensional vector, *x*. Thus, *x* is a vector field on the model grid, where each element *x_i_* represents the 11-dimensional vector defined on the model’s horizontal grid. Each index *i* uniquely identifies a grid point on the sphere, with (lon, lat) = (ϕ*_i_*, θ*_i_*). The log of the biomass data is used and is discarded if a model grid cell has a biomass less than 1.2 × 10^−3^mg Chl/m^3^ or ice cover is over 70%. The data are normalized and standardized such that all data exist in the range [0 to 1], with the mean removed and scaled to unit variance. This is done so the features (biomass and nutrient fluxes) do not become conditioned by contrasts in the ranges of possible values. The clustering should capture the variational relationships from the key probabilistic distances between features rather the geographic distances. Quantifying these distances, important features emerge while unnecessary detail is discarded. In ecological terms, this is necessary because some phytoplankton types that have little biomass can have large biogeochemical impact, e.g., diazotrophs fixing nitrogen. The covariability of these types is highlighted when the data are standardized and normalized.

The t-SNE algorithm is used to make existing similar regions stand out more clearly, by emphasizing feature proximity in the high-dimensional space in a lower-dimensional representation. Previous work aiming to build deep neural networks for remote sensing applications used t-SNE, demonstrating its skill in separating key features (*23*). This is a necessary step toward identifying robust clusters in the feature data while avoiding nonconvergent solutions (note S2). Using a Gaussian kernel, t-SNE preserves the statistical properties of the data by mapping each high-dimensional object onto a point in 3D phase space in a way that ensures a high probability of similar objects being close in both the high- and low-dimensional space (*24*). Given a set of *N* high-dimensional objects *x*_1_,..., *x_N_*, the t-SNE algorithm performs a reduction by minimizing the Kullback-Leibler (KL) divergence (*25*). The KL divergence is a measure of how different one probability distribution is from a second reference probability distribution, effectively assessing the likelihood of association between a low-dimensional rendition of the high-dimensional features. If *x_i_* is the *i*-th object and *x_j_* is the *j*-th object in the *N*-dimensional space, and *y_i_* is the *i*-th object and *y_j_* is the *j*-th object in the low-dimensional space, t-SNE defines a probability of similarity, *p*

$$p_{j|i}=\frac{\text{exp}\;(-∥x_{i}-x_{j}{∥}^{2}/2\sigma_{i}^{2})}{\sum_{k\neq i}\text{exp}\;(-∥x_{i}-x_{k}{∥}^{2}/2\sigma_{i}^{2})}$$

and the same for a reduced dimensional set

$$q_{i|j}=\frac{{\left(1+∥y_{i}-y_{j}{∥}^{2}\right)}^{-1}}{\sum_{k\neq i}{\left(1+∥y_{j}-y_{k}{∥}^{2}\right)}^{-1}}$$

The KL divergence is

$$\text{KL}(P\|Q)=\sum_{i\neq j}p_{\mathrm{ij}}\text{log}\frac{p_{\mathrm{ij}}}{q_{\mathrm{ij}}}$$

**Fig. 2:**
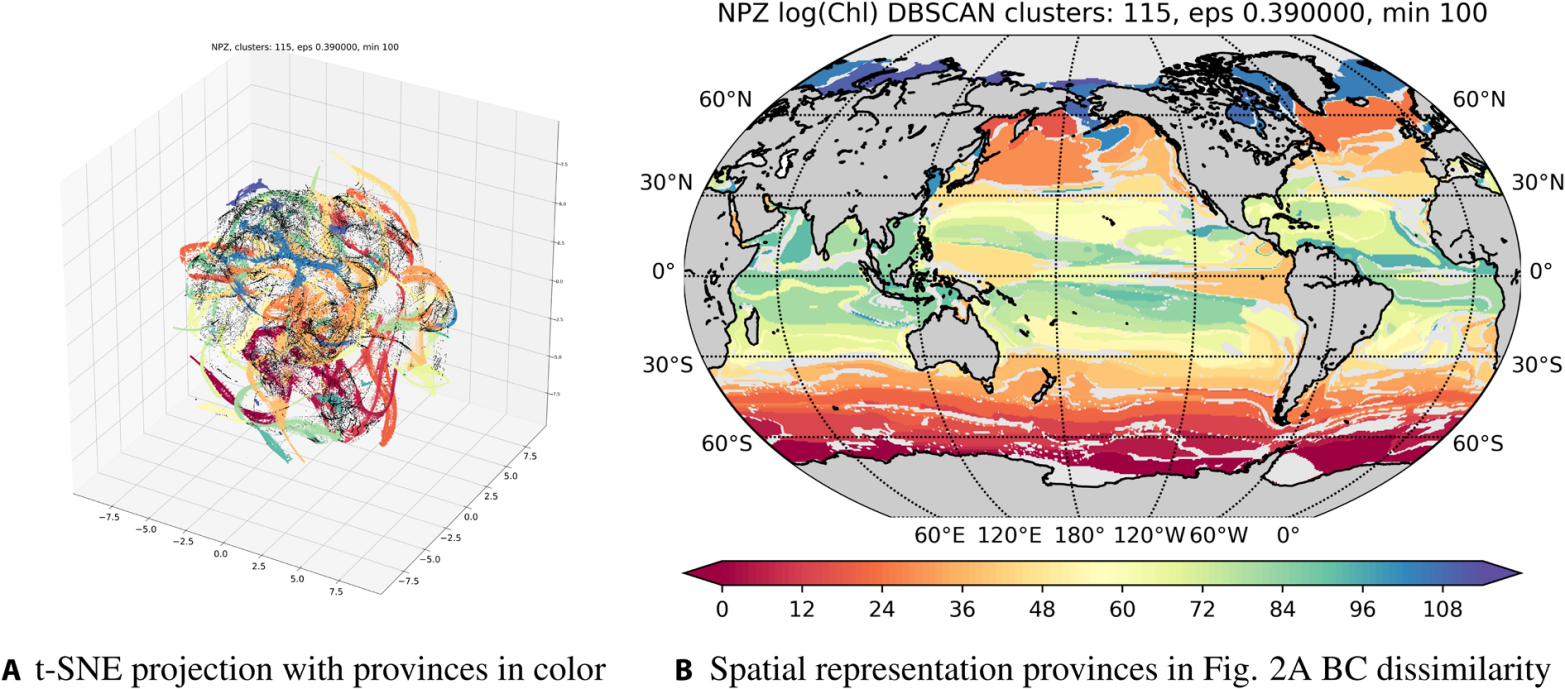
Eco-provinces in geographical and t-SNE space. (**A**) Modeled nutrient supply rates, phytoplankton, and zooplankton functional group biomass as rendered by the t-SNE algorithm and colored by province using DBSCAN. Each point represents one point in the high-dimensional space, with the majority of points captured as is demonstrated in Fig. 6B. Axes refer to “t-SNE” dimensions 1, 2, and 3. (**B**) Geographical projection of the provinces discovered by DBSCAN onto the origin latitude-longitude grid. Colors should be considered arbitrary but correspond to (A).

Figure 2A illustrates the effect of reducing the 11-dimensional combined biomass and nutrient flux vector set to 3D. The motivation for applying t-SNE can be likened to that of principal components analysis (PCA), using variance attributes to emphasize regions/properties of the data and thus reduce the dimensionality. The t-SNE method was found to be superior to PCA in delivering robust and reproducible results for the eco-provinces (see note S2). This is likely because the orthogonality assumption that underlies PCA is not appropriate for identifying key interactions between highly nonlinearly interacting features because PCA focuses on linear covariance structure (*26*). Using remotely sensed data, Lunga *et al.* (*27*) illustrate how complex and nonlinear spectral features that depart from Gaussian distributions can be highlighted using SNE methods.

### Clustering: Finding similar regions with DBSCAN

The points in the t-SNE scatterplot in Fig. 2A are each associated with a latitude and longitude. If two points are close to each other in Fig. 2A, this is because their biomass and nutrient fluxes are similar, not due to geographical proximity. The colors in Fig. 2A are the clusters found using the DBSCAN method (*28*). Looking for densely packed observations, the DBSCAN algorithm uses the distance in the 3D representation between points (ϵ = 0.39; see Materials and Methods for a discussion of this choice), and the number of similar points is needed to define a cluster (here 100 points, see above). The DBSCAN method makes no assumptions about the shapes or numbers of clusters in the data, as follows:

1) A random data point *y_i_* is selected.

2) The number of immediately neighboring points within distance ϵ of *y_i_* is measured.

3) The cluster boundary is determined repeating step 2 iteratively for all points identified as within distance ϵ. If the number of points is larger than the set minimum, it is designated as a cluster.

4) A new point is chosen at random from the remaining unclassified data, and the method is repeated.

The data that do not meet the minimum cluster member and distance ϵ metric are counted as “noise” and not assigned a color. DBSCAN is a fast and scalable algorithm, with a worst-case performance of *O*(*n*^2^), and is effectively not stochastic for the present analysis. Setting the minimum number of points was determined using expert assessment, with results not being robust within ≈±10 after adjustment of the distance ϵ. This distance was set using the degree of connectiveness (Fig. 6A) and the percentage of ocean covered (Fig. 6B). The connectiveness is defined as the resultant number of clusters and is sensitive to the ϵ parameter. A low connectiveness indicates underfitting, artificially grouping areas together. A high connectiveness indicates overfitting. A higher minimum number could conceivably be used, but arriving at a robust solution would be unlikely if the minimum exceeds ca. 135 (see Materials and Methods for further details).

### Back-projecting onto the globe

The 115 clusters identified in Fig. 2A are presented projected back onto the globe in Fig. 2B. Each color corresponds to a coherent combination of biogeochemical and ecological factors identified by DBSCAN. Once the clusters are determined, the association of each point in Fig. 2A to a specific latitude and longitude is used to project clusters back to the geographical domain. Figure 2B illustrates this with colors of clusters the same as in Fig. 2A. Similar colors should not be interpreted as ecological similarity, as they are assigned by the order in which the algorithm discovers clusters.

Regions in Fig. 2B can be seen as qualitatively similar to established regions in the physics and/or biogeochemistry of the ocean. For example, the clusters in the Southern Ocean are zonally symmetric, oligotrophic gyres emerge, sharp transitions suggest the influence of trade winds, and distinct regions associated with upwelling are seen, e.g., in the equatorial Pacific.

### Ecological similarity: Bray-Curtis dissimilarity

To understand the ecological context of the eco-provinces, the intracluster ecology is assessed using a variant on the Bray-Curtis (BC) dissimilarity metric (*29*). The BC metric is a statistic used to quantify the community structure dissimilarity between two different sites. The BC metric is applied to the biomass of the 51 types of phyto- and zooplankton

$$BC_{n_{i}n_{j}}=1-\frac{2C_{n_{i}n_{j}}}{S_{n_{i}}+S_{n_{j}}}$$

*BC_n_i_n_j__* refers to the dissimilarity of assemblage *n_i_* compared to assemblage *n_j_*, where *C_n_i_n_j__* is the minimum of biomass of individual types present in both assemblages *n_i_* and *n_j_*, while *S_n_i__* refers to the sum over all the biomass present in both assemblages *n_i_* and *S_n_j__*. The BC dissimilarity is similar to a distance metric but operates in a non-Euclidean space, which is likely better suited to ecological data and its interpretation.

**Fig. 3:**
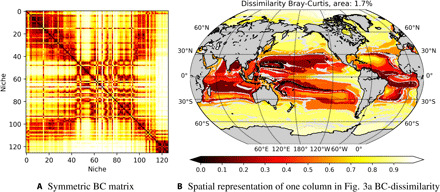
The eco-province BC dissimilarity. (**A**) BC dissimilarity metric evaluated for every province compared to every other for the global surface 20-year mean of the 51 plankton biomasses. Note the expected symmetry of the values. (**B**) Spatial projection of one column (or row). The global distribution of BC dissimilarity metric evaluated for a province in the oligotrophic gyre compared to every other for the global surface 20-year mean. Black (BC = 0) denotes an identical region, while white (BC = 1) denotes no similarity.

For each cluster identified in Fig. 2B, the intra- and interprovince BC dissimilarity can be assessed. The intraprovince BC dissimilarity refers to the dissimilarity between the province mean and each point in it. The interprovince BC dissimilarity refers to how similar one province is to each other province. Figure 3A illustrates the symmetric BC matrix (0, black: perfect correspondence; 1, white: completely dissimilar). Each line in this plot demonstrates patterns in the data. Figure 3B demonstrates the geographical implications of the BC results from Fig. 3A for individual provinces. For a province in the low nutrient oligotrophic region, Fig. 3B demonstrates that large areas are reasonably similar symmetrically around the equator and in the Indian Ocean, but the higher latitudes and upwelling regions are markedly different.

**Fig. 4:**
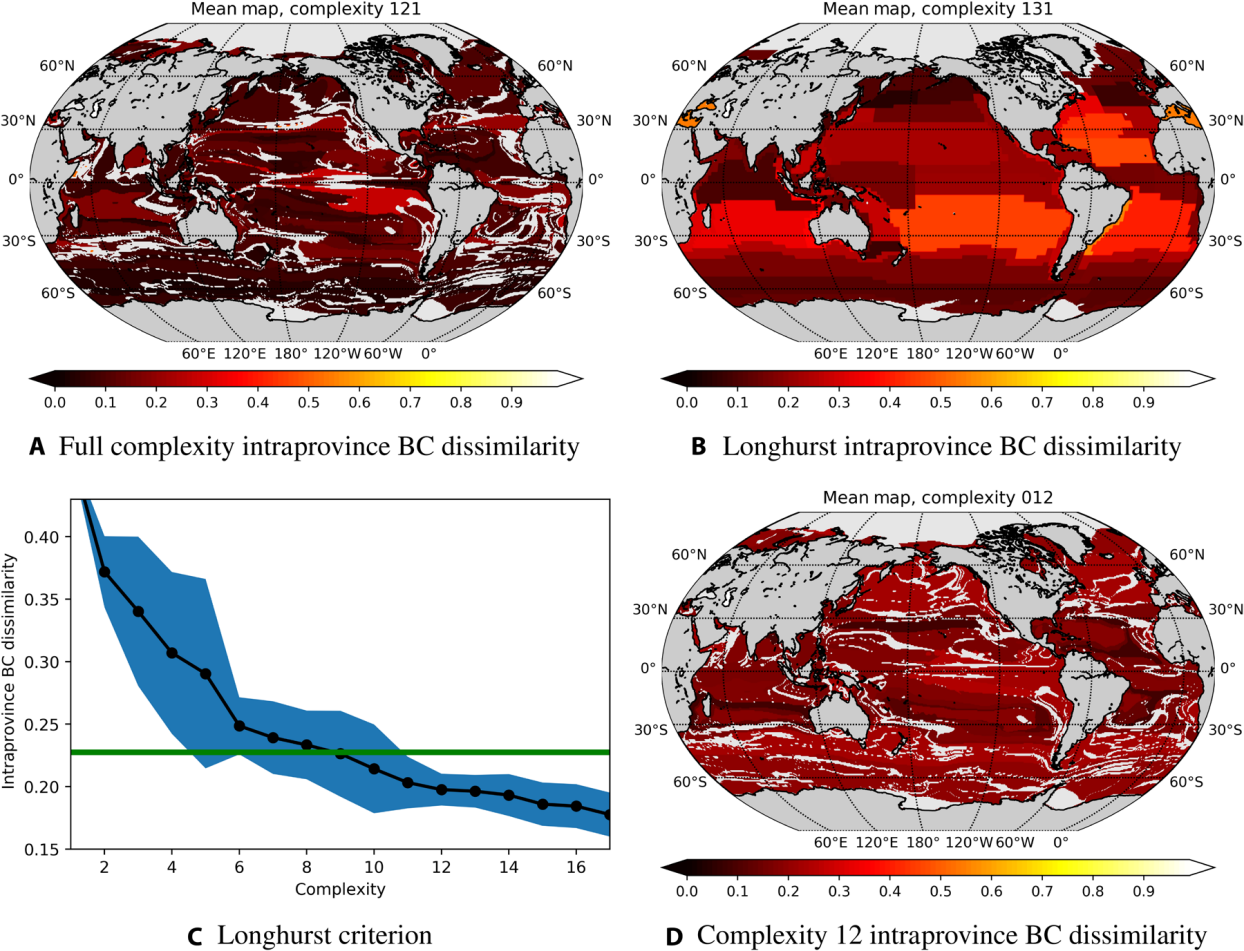
Heuristic processes to determine a minimum level of biogeochemical complexity. (**A**, **B**, and **D**) The intraprovince BC dissimilarity is assessed as the mean BC dissimilarity of the individual grid point communities compared to the mean province with no reduction in complexity. (B) The global mean intraprovince BC dissimilarity is 0.227 ±0.117. This is the benchmark for the ecologically motivated sorting presented in this work [green line in (C)]. (**C**) Averaged intraprovince BC dissimilarity: The black line illustrates the intraprovince BC dissimilarity of increasing complexity. The 2σ is from 10 repeats of the eco-province recognition process. For the full complexity in the provinces discovered by DBSCAN, (A) illustrates that an intraprovince BC dissimilarity of 0.099 is reached, while sorting into a complexity of 12 as suggested by (C) results in an intraprovince BC dissimilarity of 0.200, as demonstrated in (D).

The intraprovince BC dissimilarity within each province from Fig. 2B is illustrated in Fig. 4A. Determined using the mean area averaged assemblage within one cluster, and determining the BC dissimilarity of each grid point within the province to the mean, it illustrates how well the SAGE method is able to separate the 51 types of the model data according to ecological similarity. The global mean intracluster BC dissimilarity for all 51 types is 0.102 ±0.0049.

The equivalent Longhurst intraprovince BC dissimilarity is presented in Fig. 4B using the biomass of the 51 plankton types, with a global mean across provinces of 0.227, and a standard deviation across grid points referenced to the province BC dissimilarity of 0.046. This is larger than for the clusters identified in Fig. 1B. Using the sum of the seven functional groups instead, the mean intraseasonal BC dissimilarity of the Longhurst provinces increases to 0.232.

The maps of the global eco-provinces offer intricate detail of ecological interactions that are unique and offer a refinement in terms of ecosystem structure over using Longhurst provinces. The eco-provinces are anticipated to provide insight into the processes controlling the numerical model ecosystem, and such insights could assist exploration of in situ efforts. For the purpose of this study, the over hundred provinces cannot be adequately showcased. The following section presents the SAGE method to aggregate provinces.

### Defining AEPs

One of the uses of provinces is to facilitate understanding of where they are and how they are governed. To identify emergent properties, the method in Fig. 1B illustrates the nesting of ecologically similar provinces. Eco-provinces are grouped together in terms of their ecological similarity, and this grouping of provinces is called AEPs. An adjustable level of “complexity” is set in terms of the number of aggregated provinces that will be considered. The term complexity is used as it allows the level of the emergent properties to be adjusted. For defining meaningful aggregation, the mean intraprovince BC dissimilarity from the Longhurst provinces of 0.227 is used as a benchmark below which the aggregated provinces are no longer considered useful.

The eco-provinces are coherent across the globe, as demonstrated in Fig. 3B. Some configurations are very “common,” as seen using the interprovince BC dissimilarity. Inspired by methods from genetics and graph theory, “connectivity graphs” are used to sort the >100 provinces according to which province they are most similar to. The metric of “connectivity” here is determined using the interprovince BC dissimilarity (*30*). The number of spatially larger provinces that the >100 provinces can be sorted into is here referred to as the complexity. The AEPs are the product of sorting the full >100 provinces into this subset of the most dominant/highly connected eco-provinces; each eco-province is assigned to the dominant/highly connected eco-province they are most similar to. This aggregation determined by the BC dissimilarity allows a nested approach to global ecology.

The chosen complexity can be anything from 1 to the full complexity from Fig. 2A. At low complexities, the AEPs can become degenerate because of the probabilistic dimensionality-reduction step (t-SNE). Degeneracy implies that the eco-provinces could be assigned to different AEPs between iterations, changing the geographical area covered. Fig. 4C illustrates the spread of the intraprovince BC dissimilarity in the AEPs of increasing complexity across 10 realizations (illustration in Fig. 1B). In Fig. 4C, 2σ (blue area) is a measure of the degeneracy within the 10 realizations, and the green line represents the Longhurst benchmark. A complexity of 12 is demonstrated to keep the intraprovince BC dissimilarity both below the Longhurst benchmark in all realizations and a relatively small 2σ degeneracy. In sum, the minimum recommended complexity is 12 AEPs, for which the mean intraprovince BC dissimilarity assessed using the 51 plankton types is 0.198 ±0.013, as seen in Fig. 4D. Using the sum of the seven plankton functional groups, the mean intraprovince BC dissimilarity 2σ is instead 0.198 ±0.004. The comparison between the BC dissimilarity computed with either the seven functional group summed biomass or the full 51 plankton types biomasses suggests that the SAGE method is appropriate for the 51-dimensional case, although it was trained on the biomass sum of the seven functional groups.

Depending on the purpose of any study, a different level of complexity could be considered. A regional study might want the full complexity (i.e., all 115 provinces). As an example and for clarity, the lowest recommended complexity 12 is considered.

### Utility of AEPs: Community structure and their controls

**Fig. 5:**
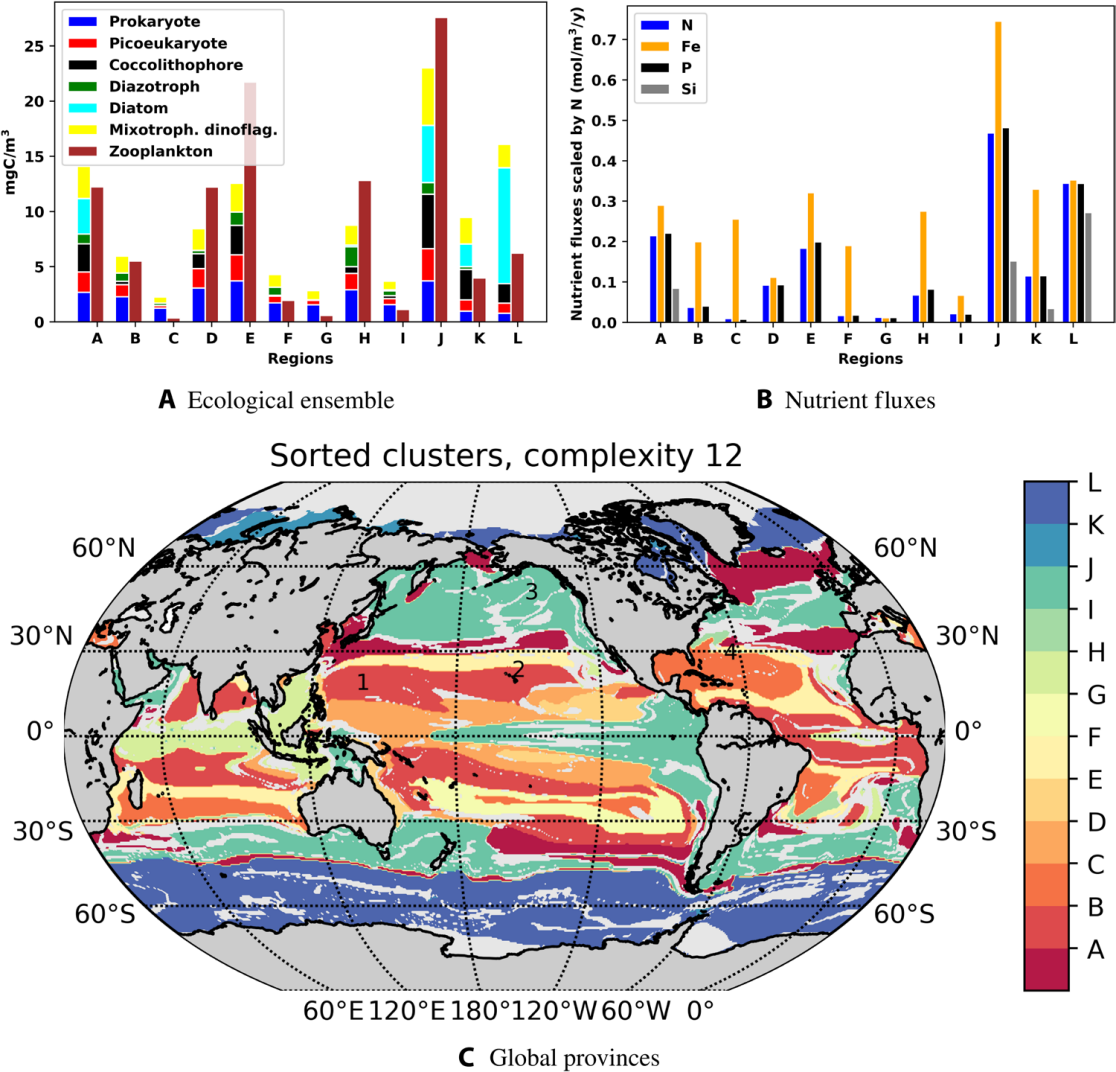
AEP interpretation for complexity 12. Sorting the provinces into the 12 AEPs A to L. (**A**) Biomass (mgC/m^3^) of the ecological ensemble in the 12 provinces. (**B**) Nutrient flux rates (mmol/m^3^ per year) for dissolved inorganic nitrogen (N), iron (Fe), phosphate (P), and silicic acid (Si). Fe and P are multiplied by 16 and 16×10^3^, respectively, so that the bars are normalized to the phytoplankton stoichiometric requirements. (**C**) Note the distinction between polar, subtropical gyres and dominantly seasonal/upwelling regions. Monitoring stations are marked as follows: 1, SEATS; 2, ALOHA; 3, station P; and 4, BATS.

As an example of the utility of the SAGE method, here, the minimum complexity 12 AEPs are used to explore the controls on the emergent community structure. Figure 5 illustrates the ecological insights grouped by AEPs (named A to L): The geographical extent (Fig. 5C), functional group biomass composition (Fig. 5A), and nutrient supply (Fig. 5B) scaled by N in the stoichiometric Redfield ratio (N:Si:P:Fe, 1:1:16:16 ×10^3^) are shown. For this latter panel, P is multiplied by 16 and Fe by 16×10^3^,so the bars are comparable to the phytoplankton nutrient requirements.

The identified AEPs are unique. There is some symmetry around the equator in the Atlantic and Pacific oceans, and similar but augmented regions exist in the Indian Ocean. Some AEPs hug the western sides of continents associated with upwelling regions. The Antarctic circumpolar current is seen as a large zonal feature. The subtropical gyres stand out as complex series of oligotrophic AEPs. The familiar patterns of differences in biomass between picoplankton dominated oligotrophic gyres and diatom rich polar regions are apparent in these provinces.

AEPs with very similar total phytoplankton biomass can have very different community structure and cover very different geographical areas, such as D, H, and K, which have similar total phytoplankton biomass. AEP H is present mainly in the equatorial Indian Ocean and has a larger population of diazotrophs. AEP D is found in several basins, but is prominent in the Pacific surrounding the very highly productive region around the equatorial upwelling. The shape of this province in the Pacific is reminiscent of planetary wave trains. AEP D has very few diazotrophs but more coccolithophores. AEP K is found only in the high Arctic Ocean and has more diatoms and fewer picoplankton than the other two provinces. It is notable that zooplankton biomass in the three regions is also very different, with AEP K having relatively low zooplankton abundance, but AEP D and H having relatively similar, higher, levels. Thus, although their biomass (and hence also Chl-a) is similar, these provinces are very different: Chl-based province detection would likely not capture these differences.

It is also apparent that some AEPs that have very different biomass can be similar in terms of their phytoplankton community structure. This is seen in AEP D and E for example. These are close to each other, in the Pacific, where AEP E is close to the highly productive AEP J. Again, there is not a clear connection between phytoplankton biomass and zooplankton abundance.

The AEPs can be understood in terms of the nutrient supplies to them (Fig. 5B). Diatoms only exist where there is sufficient silicic acid supply; generally, the higher the silicic acid supply, the higher the diatom biomass. Diatoms are seen in AEPs A, J, K, and L. The proportion of diatom biomass relative to other phytoplankton is dictated by how much N, P, and Fe are supplied relative to the diatoms demands. For instance, AEP L is dominated by diatoms and has the highest supply of Si relative to the other nutrients. In contrast, though more productive, AEP J has fewer diatoms and less Si supply (both total and relative to the other nutrients).

Diazotrophs have the ability to fix N, but also grow slowly (*31*). They coexist with other phytoplankton where there is an excess of Fe and P relative to the demands of the nondiazotrophs (*20*, *21*). It is notable that there is higher diazotroph biomass, where the amount of Fe and P supply is relatively large relative to the N supply. In this manner, the diazotroph biomass is larger in AEP H than in J, although the overall biomass in AEP J is higher. Note that AEP J and H are very different geographically, with H located in the equatorial Indian Ocean.

The insight gained from patterns in the minimum complexity of 12 AEPs would be much less clear if the unique ecosystem structure was not separated into provinces. SAGE-generated AEPs facilitate the coherent and simultaneous comparison of the complicated and high-dimensional information from the ecosystem model. The AEPs effectively highlight why and where Chl is not a good proxy for determining community structure or abundance of zooplankton in higher trophic levels. A detailed analysis of the topic of an ongoing study is beyond the scope of this paper. The SAGE method provides a way to explore other mechanisms in the model in a more tractable way than looking from point to point.

## DISCUSSION AND CONCLUSION

The SAGE method is presented, designed to help elucidate the overwhelmingly complicated ecological data from a global physical/biogeochemical/ecosystem numerical model. Eco-provinces are determined by summation of biomass across plankton functional groups, application of the t-SNE probabilistic dimensionality reduction algorithm, and clustering using the unsupervised ML method DBSCAN. An interprovince BC dissimilarity/graph theory method for nesting is applied to arrive at robust AEPs useful for global interpretation. Both the eco-provinces and AEPs are unique by construction. The AEP nesting can be adjusted between the full complexity of the original eco-provinces and the minimum recommended threshold of 12 AEPs. The nesting and determination of a minimal complexity for AEPs are seen as a crucial step, as the probabilistic t-SNE makes the <12 complexity AEPs degenerate. The SAGE method is global and spans a complexity range from >100 AEPs to 12. For simplicity, the present focus is on the complexity 12 global AEPs. Future studies, particularly regional ones, could find a smaller spatial subset within the global eco-provinces useful and potentially perform the aggregation within such a smaller region to leverage the same ecological insight as is discussed here. Suggestions are offered regarding how these eco-provinces, and insight gained from them, could be used for further ecological understanding, facilitate model intercomparison, and potentially improve monitoring of marine ecosystems.

The eco-province and AEPs that the SAGE method identified are based on data from a numerical model. Numerical models are, by definition, simplified constructs that attempt to capture the essence of a target system, and different models can vary in their plankton distributions. The numerical model used in this study does not fully capture some of the patterns observed (e.g., in Chl estimates of the equatorial regions and Southern Ocean). Capturing only a fraction of the diversity in the real ocean, and not resolving the meso- and sub-mesoscale, likely affects nutrient fluxes and smaller-scale community structures. Despite these shortcomings, AEPs are shown to be useful in helping to understand the complex model. The AEPs offer a potential numerical model intercomparison tool, by assessing where similar ecological provinces are found. The present numerical model captures gross patterns of remotely sensed phytoplankton Chl-a concentrations and distributions of plankton size and functional groups (note S1 and fig. S1) (*2*, *32*).

The AEPs fit into oligotrophic versus mesotrophic regions as indicated by the 0.1 mgChl-a/m^−3^ contour (fig. S1B): AEPs B, C, D, E, F, and G are oligotrophic, and the remainder are in regions of higher Chl-a. The AEPs show some correspondence to the Longhurst provinces (fig. S3A), for example, the Southern Ocean and equatorial Pacific. In some regions, the AEPs cover several Longhurst regions and vice versa. Because the intent of the delineation of provinces here and in Longhurst is not the same, differences are anticipated. Multiple AEPs within a single Longhurst province suggest that some regions with similar biogeochemistry may have very different ecosystem structure. The AEPs show some correspondence to physical regimes as revealed using unsupervised learning (*19*), such as in high upwelling regimes (e.g., Southern Ocean and the equatorial Pacific; fig. S3, C and D). Such correspondences suggest where plankton community structure is strongly influenced by ocean dynamics. In regions such as the North Atlantic, AEPs cross through physical provinces. Mechanisms causing these discrepancies could include processes such as dust delivery, leading to very different nutrient regimes even within a similar physical regime.

The eco-provinces and AEPs suggest that using Chl alone is not able to identify ecological composition, as is already appreciated by the marine ecological community. This is seen in AEPs with similar biomass but markedly different ecological composition (e.g., D and E). In contrast, AEPs such as D and K have very different biomass but similar ecological composition. The AEPs emphasize that the relationship between biomass, ecological composition, and zooplankton abundance is complex. For example, while AEP J stands out in terms of both high phytoplankton and zooplankton biomass, AEP’s A and L have similar phytoplankton biomass but A has much higher zooplankton abundance. The AEPs highlight that phytoplankton biomass (or Chl) cannot be used to predict zooplankton biomass. Zooplankton is the base of the food chain for fisheries, and more accurate estimates could lead to better resource management. Future ocean color satellites [e.g., PACE (plankton, aerosol, cloud and ocean ecosystem)] might be better positioned to help estimate phytoplankton community structure. Using AEP predictions, estimates of zooplankton from space could potentially be facilitated. Methods like SAGE, together with new technology, as well as the increasing in situ data available (e.g. Tara and follow-on studies) for ground truthing, could together provide a step toward satellite-based monitoring of the health of an ecosystem.

The SAGE method provides a convenient way to assess some of the mechanisms that control the features in the provinces, e.g., biomass/Chl, net primary production, and community structure. For example, the relative amount of diatoms is set by the imbalance in the Si to N, P, and Fe supplies relative to the phytoplankton stoichiometric requirements. With balanced supply rates, communities are diatom dominated (L), and where supply rates are less balanced (i.e., with lower Si supply relative to the diatoms nutrient demands), diatoms comprise only a smaller fraction (K). Diazotrophs thrive where the Fe and P supplies are in excess of the N supplies (e.g., E and H). Explorations of controlling mechanisms are made substantially more useful through the context provided by AEPs.

The eco-provinces and AEPs are regions of similar community structures. A time series from a location within one eco-province or AEP could be seen as a point of reference and a representative of the area covered by the eco-province or AEP. Long-term in situ monitoring stations offer such time series. Long-term in situ datasets will continue to be invaluable, and the SAGE method could be seen as a method to help determine locations where new sites would be most useful from the perspective of monitoring community structure. For example, the time series from a long-term oligotrophic habitat assessment (ALOHA) is in AEP B (Fig. 5C, label 2) in an oligotrophic region. Because ALOHA is close to the boundary to another AEP, the time series may not be representative of the whole region, as previously suggested (*33*). Within the same AEP B, the time series SEATS (southeast asia time-series) is southwest of Taiwan (*34*), further from the boundaries of other AEPs (Fig. 5C, label 1), and could serve as a better location within which to monitor AEP B. The BATS (bermuda atlantic time-series study) time series in AEP C (Fig. 5C, label 4) is very close to the border of AEPs C and F, suggesting that monitoring AEP C using the BATS time series directly could be problematic. The P station (Fig. 5C, label 3) in AEP J is quite far from an AEP boundary and could therefore be more representative. The eco-provinces and AEPs could help establish a monitoring framework suitable for assessing global change, as the provinces allow assessment of where in situ sampling could offer key insight. The SAGE method can be developed further for application to climatological data to assess temporal province variability.

The success of the SAGE method is achieved through careful application of data science/ML methods, together with domain-specific knowledge. Specifically, dimensionality reduction is performed using t-SNE, retaining the covariance structure of the high dimensional data, and facilitating visualizing the covariance topology. The data are arranged in streaks and sheets of covariance (Fig. 2A), indicating that purely distance-based metrics such as *K*-means are inappropriate as they often assume a Gaussian (round) underlying distribution (discussed in note S2). The DBSCAN method is appropriate for arbitrary covariance topologies, offering robust identification provided that careful attention is given to setting parameters. The t-SNE algorithm is computationally costly, limiting its present application to larger data volumes, meaning that application to depth- or time-varying fields is difficult. Work on the scalability of t-SNE is ongoing. The t-SNE algorithm has the potential to scale well in the future, as the KL distance is readily parallelizable (*35*). Alternative promising methods of dimensionality reduction that, to date, scale better include the uniform manifold approximation and projection (UMAP) technique, but evaluation in the context of oceanographic data is necessary. Implications of better scalability would be classification, e.g., over the mixed layer, for global climatologies or models with varying complexity. The regions that fail to be classified within any province by SAGE can be seen as the remaining black dots in Fig. 2A. Geographically, these regions are largely in highly seasonal areas, suggesting that capturing the time-evolving eco-provinces would provide better coverage.

To construct the SAGE method, ideas from complex systems/data science have been leveraged, exploiting the ability to determine clusters of functional groups (high probability of close proximity in an 11-dimensional space) and determine provinces. These provinces delineate a specific volume in our 3D t-SNE phase space. Similarly, Poincaré sections can be used to assess the “volume” of state space occupied by a trajectory to determine “regular” or “chaotic” behavior (*36*). For the static 11-dimensional model output, the volume occupied after data are cast into a 3D phase space could be interpreted similarly. The relation between geographical area and the area in 3D phase space is not simple but can be interpreted in terms of ecological similarity. The more conventional BC dissimilarity metric was preferred for this reason.

Future work will repeat the SAGE method for seasonally varying data to assess the spatial variability in the identified provinces and AEPs. A future goal is to leverage this method to help determine which provinces could be determined by satellite measurements such as Chl-a, remotely sensed reflectance, and sea-surface temperature. This would allow remote sensing assessments of ecological composition and highly agile monitoring of the eco-provinces and their variability.

## MATERIALS AND METHODS

The purpose of this study is to present the SAGE method for defining eco-provinces by their distinct plankton community structure. Here, more detail is provided on the physical/biogeochemical/ecosystem model, as well on parameter selection for t-SNE and DBSCAN algorithms.

### Model framework

The physical component of the model comes from the Estimating the Circulation and Climate of the Ocean [ECCOv4; (*37*)] global state estimate described by (*38*). The state estimate has a nominally 1^∘^ resolution. A least-squares with Lagrange multipliers approach is used to obtain observationally adjusted initial and boundary conditions as well as internal model parameters, resulting in a free-running version of the MIT General Circulation Model (MITgcm) (*39*) that is optimized to track observations.

The biogeochemical/ecosystem is described more fully (i.e., equations and parameter values) in (*2*). The model captures the cycling of C, N, P, Si, and Fe through inorganic and organic pools. The version used here includes 35 phytoplankton: 2 pico-prokaryotes and 2 pico-eukaryotes (that are adapted to low nutrient environments), 5 coccolithophores (that have calcium carbonate coverings), 5 diazotrophs (that fix nitrogen gas and thus are not limited by availability of dissolved inorganic nitrogen), 11 diatoms (that form siliceous coverings), 10 mixotrophic dinoflagellates (that can both photosynthesize and graze other plankton), and 16 zooplankton (which graze on other plankton). These are referred to as “biogeochemical functional groups,” as they affect the biogeochemistry of the ocean differently (*40*, *41*) and are frequently used in observational and modeling studies. In this model, each functional group is made up of several plankton of different sizes spanning 0.6 to 2500 μm equivalent spherical diameter.

Parameters influencing phytoplankton growth, grazing, and sinking are related to size, with specific differences between the six phytoplankton functional groups (*32*). Results from this 51 plankton component of the model have been used in several recent studies (*42*–*44*), though in a different physical framework.

The coupled physical/biogeochemical/ecosystem model was run for 20 years from 1992 to 2011. Output from the model includes the plankton biomass, nutrient concentrations, and rate of supply of the nutrients (DIN, PO4, Si, and Fe). For this study, the 20-year mean of these outputs was used as the input for the eco-provinces. Distribution of Chl, plankton biomass, and nutrient concentrations, as well as distributions of functional groups compare well with satellite and in situ observations [see (*2*, *44*), note S1, and figs. S1 to S3].

### Parameter selection for t-SNE and DBSCAN

For the SAGE method, the main source of stochasticity comes from the t-SNE step. Stochasticity can hinder reproducibility, meaning that results are not robust. The SAGE method uses a stringent test of robustness, by identifying one set of parameters for t-SNE and DBSCAN that consistently identify clusters when repeated. Determining t-SNE parameter “perplexity” can be understood as determining the degree to which the mapping from high to low dimensionality should respect local or more global features of the data. A perplexity of 400 and 300 iterations was arrived at.

**Fig. 6:**
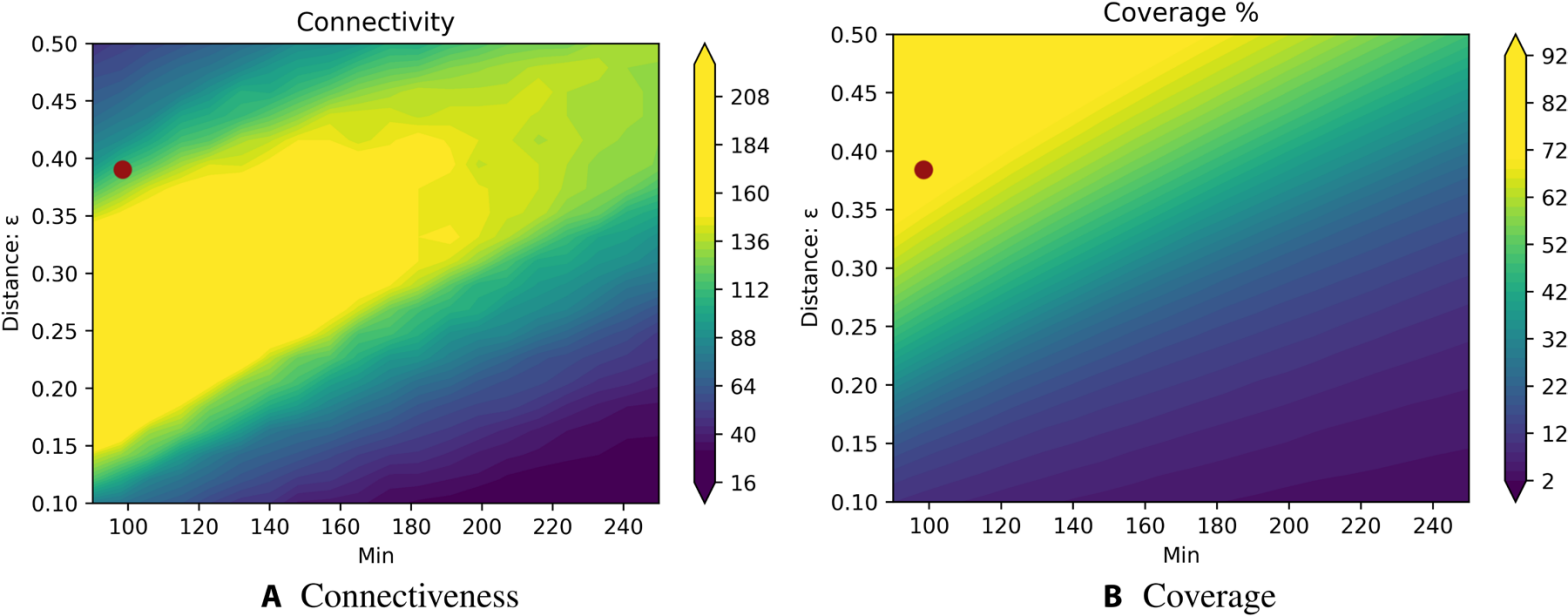
Setting the DBSCAN parameters. Setting the parameters for t-SNE, the resultant number of found clusters is used as a measure of the connectiveness (**A**) and the percentage of the data assigned to a cluster (**B**). The red dot illustrates the optimal combination of coverage and connectedness. The minimum number was set on the basis of minimum number relevant for ecology.

For the clustering algorithm DBSCAN, the minimum size of the data points within a cluster and the distance metric ϵ need to be determined. The minimum number is set using expert guidance with knowledge of what is appropriate for the present numerical modeling framework and resolution; a minimum number of 100 was set. A higher minimum number (ca. <135, before the upper band of green widens) could conceivably be used but would not be able to act as a substitute for the aggregation method based on the BC dissimilarity. The degree of connectiveness (Fig. 6A) is used to set the ϵ parameter, favoring a higher coverage (Fig. 6B). The connectiveness is defined as the resultant number of clusters and is sensitive to the ϵ parameter. A low connectiveness indicates underfitting, artificially grouping areas together. A high connectiveness indicates overfitting. Overfitting is also problematic because it indicates that the initial stochastic guess can lead to results that are not reproducible. Between these two extremes, there is a drastic increase (often referred to as an “elbow”), indicating the optimal ϵ. In Fig. 6A, a sharp increase is seen to a plateau (yellow, >200 clusters), followed by a sharp decrease (green, 100 clusters) up to a minimum of ca 130, surrounded by regions of very few clusters (blue, <60 clusters). In the blue regions for a minimum of 100, either one cluster largely dominates the whole ocean (ϵ < 0.42) or most of the ocean is not classified and is deemed as noise (ϵ > 0.99). The yellow region has a highly variable, nonreproducible, cluster distribution, with increasing noise as ϵ is reduced. The green region of sharp increase is referred to as the elbow. This is the optimal region, where robust clusters can be identified, as determined using the intraprovince BC dissimilarity, despite the probabilistic t-SNE. Using Fig. 6 (A and B), ϵ was set to 0.39. With a larger minimum number, arriving at an ϵ that allows robust classification would be unlikely, with values >135 seen to have a wider green region. The widening of this region suggests that the elbow will be more difficult to find or absent.

## Supplementary Material

aay4740_SM.pdf

## SUPPLEMENTARY MATERIALS

Supplementary material for this article is available at http://advances.sciencemag.org/cgi/content/full/6/22/eaay4740/DC1
