# Supplementary material for: Elucidating ecological complexity: Unsupervised learning determines global marine eco-provinces
Source: Sci Adv. 2020 May 29;6(22):eaay4740. doi: 10.1126/sciadv.aay4740 (PMC7259926; doi:10.1126/sciadv.aay4740)
Supplement: aay4740_SM.pdf [file aay4740_SM.pdf]

Science Advances
AAAS

advances.sciencemag.org/cgi/content/full/6/22/eaay4740/DC1

Supplementary Materials for

Elucidating ecological complexity: Unsupervised learning determines global marine eco-provinces

Maike Sonnewald*, Stephanie Dutkiewicz, Christopher Hill, Gael Forget

*Corresponding author. Email: maike_s@mit.edu

Published 29 May 2020, *Sci. Adv.* **6**, eaay4740 (2020)
DOI: 10.1126/sciadv.aay4740

This PDF file includes:

Notes S1 and S2
Figs. S1 to S4
References

Supplementary Materials

Note S1: Model Evaluation

The ecosystem model has been used in various ecological configuration and within different physical frameworks (*2, 32, 42, 43*). These studies have provided extensive evaluation against satellite and available in situ data. An evaluation is presented of the model output used in this study against satellite Chl-a, and against the in situ compilation of phytoplankton functional group biomass from the MAREDAT data-set (*40*).

Comparing the model annual climatological surface (0-10m) Chl-a, to Ocean Colour Climate Change Initiative project (OC-CCI, 1998-2015) estimates of Chl-a (Fig. S1), the model is seen to capture the patterns of high Chl-a in both subpolar and equatorial upwelling regions, and captures low Chl-a in subtropical gyres. Only data for regions with full annual coverage are shown (optical satellite sensors do not capture a signal in the polar winters). Note that the satellite estimates have non-negligible uncertainties associated with them (e.g. estimates have more than 35% errors (*46*)). The spatial resolution of the Darwin model does not capture important physical processes near coastlines, and lack of sedimentary and terrestrial supplies of nutrients and organic matter lead to Chl-a being too low in these regions. Chl-a is under-estimated by the model in the subtropical gyres, likely due to lack of mesoscale processes in the model that would supply additional nutrients in these regions (see e.g. (*47*)). The model Chl-a is higher than the satellite estimates in the Southern Ocean. There are likely regional biases in the satellite algorithms, these are potentially enhanced in Southern Ocean signals e.g. (*48, 49*). The model is also higher in the equatorial Pacific, likely due to insufficient iron limitation in this region. The model underestimates the Chl-a in the Atlantic Equatorial region.

The numerical model functional group distribution is compared to the latest compilation of observations (Fig. S2, (*40*), and references therein). Observations are sparse both temporally and spatially, and were averaged into 5 degree bins to facilitate visual comparison. Visual evaluation suggests that even with the spatial and temporal data taken into account overall features are captured: the ubiquitous nature of the pico-phytoplankton, the limited domain of the diazotrophs (including observed lack of diazotrophs in the South Pacific gyre), the pattern of enhanced diatom biomass in high latitudes, and low biomass in subtropical gyres. The model underestimates diazotrophy in the western equatorial Atlantic, possibly due to lack of riverine influx of nutrients/organic matter in this region. Coccolithophore biomass is overestimated relative to MAREDAT in many regions, but note that the conversion from cells to biomass in that compilation was estimated to have uncertainties of several 100% (*50*).

Note S2: PCA and k-means methods in the presence of non-Gaussian covariance structures

The interactions between the types and nutrient fluxes in the feature vector in this study are highly non-linear, implying an underlying distribution of the covariance structures are not Gaussian. The ultimate goal of clustering algorithms is to arrive at a statistical model that approximates the ”true” model from which the available data has been drawn. The underlying distribution of the data’s covariance structure has implications for which clustering algorithm is appropriate, because these are designed to ”identify” different types of underlying distributions. A Gaussian covariance would manifest as a ”round” shape. A more complex distribution will have a correspondingly complex ”shape”. Most clustering algorithms are well suited for data with an underlying Gaussian covariance distribution, but a highly non-Gaussian distribution of-

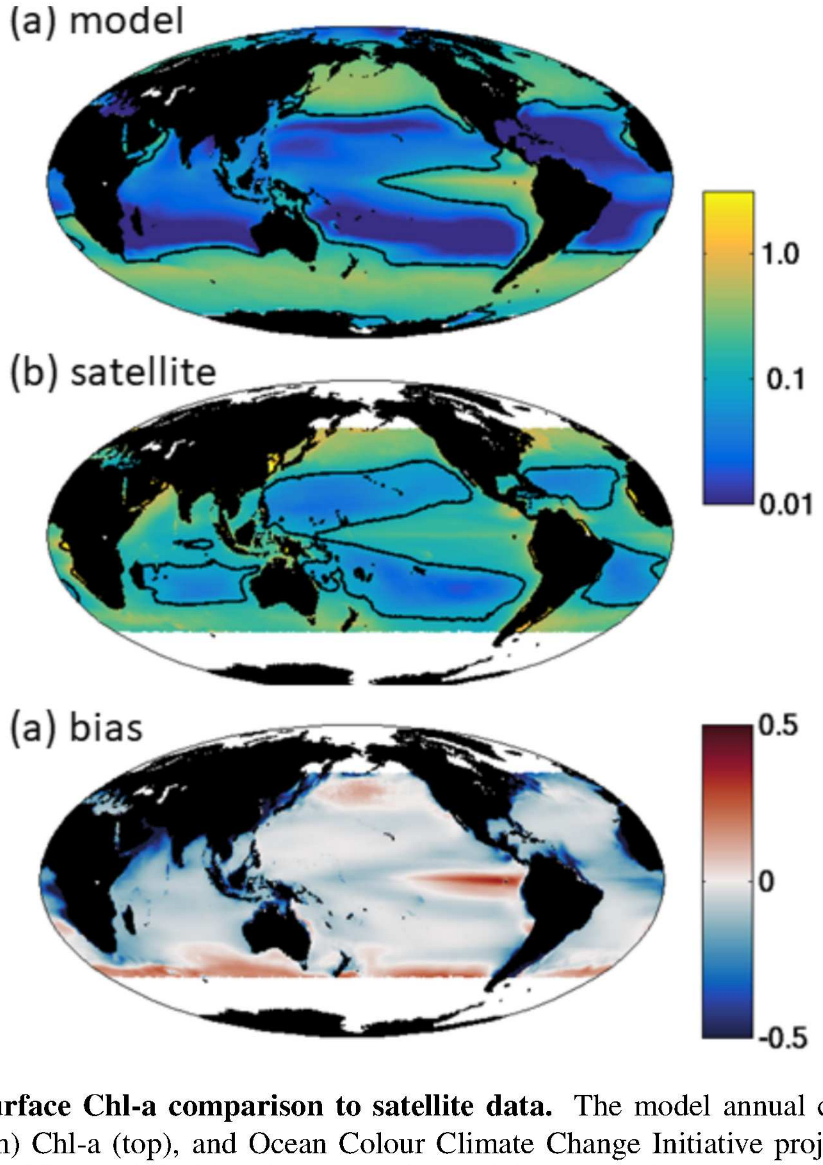

S1: Model surface Chl-a comparison to satellite data. The model annual climatological surface (0-10m) Chl-a (top), and Ocean Colour Climate Change Initiative project (OC-CCI, 1998-2015) (middle), and the bias (bottom). The model captures the patterns of high Chl-a in the subpolar regions and along the equatorial upwelling and low Chl-a in the subtropical gyres. Contours indicates 0.1 mg Chl/m^{-3}

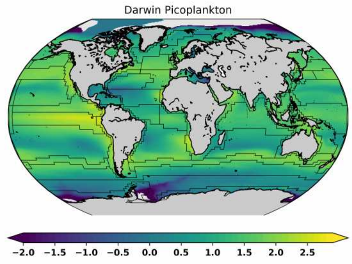

(a) Pico

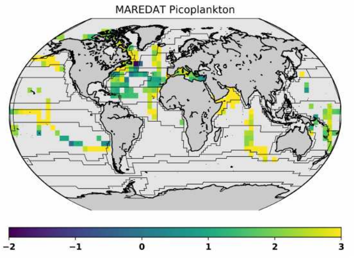

(b) Pico MAREDAT

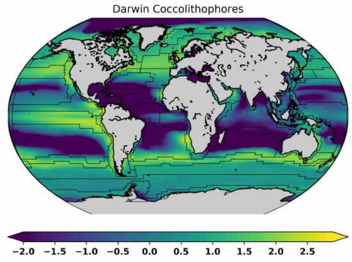

(c) Cocco

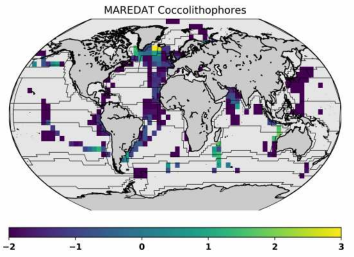

(d) Cocco MAREDAT

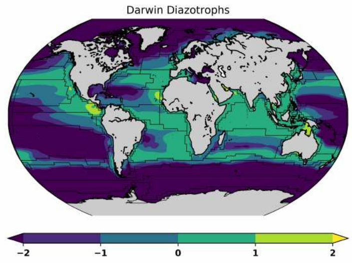

(e) Diaz

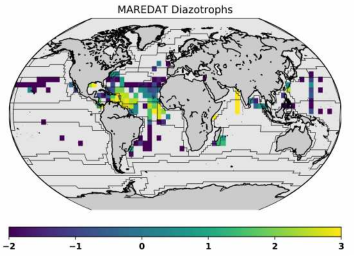

(f) Diaz MAREDAT

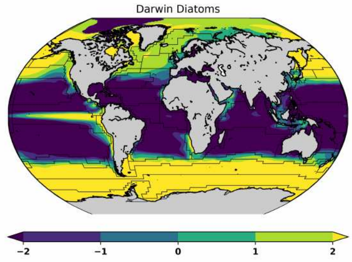

(g) Diatom

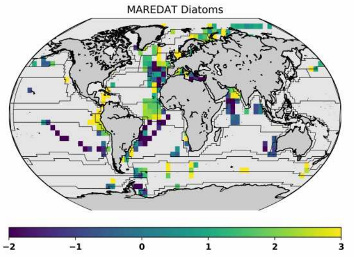

(h) Diatom MAREDAT

S2: Comparison of model and observational phytoplankton functional group biomass. Phytoplankton functional group biomass (mg C/m^{-3}) from the numerical model (a, c, e, g) and MAREDAT (b, d, f, h) (*40*), biomass on log scale.

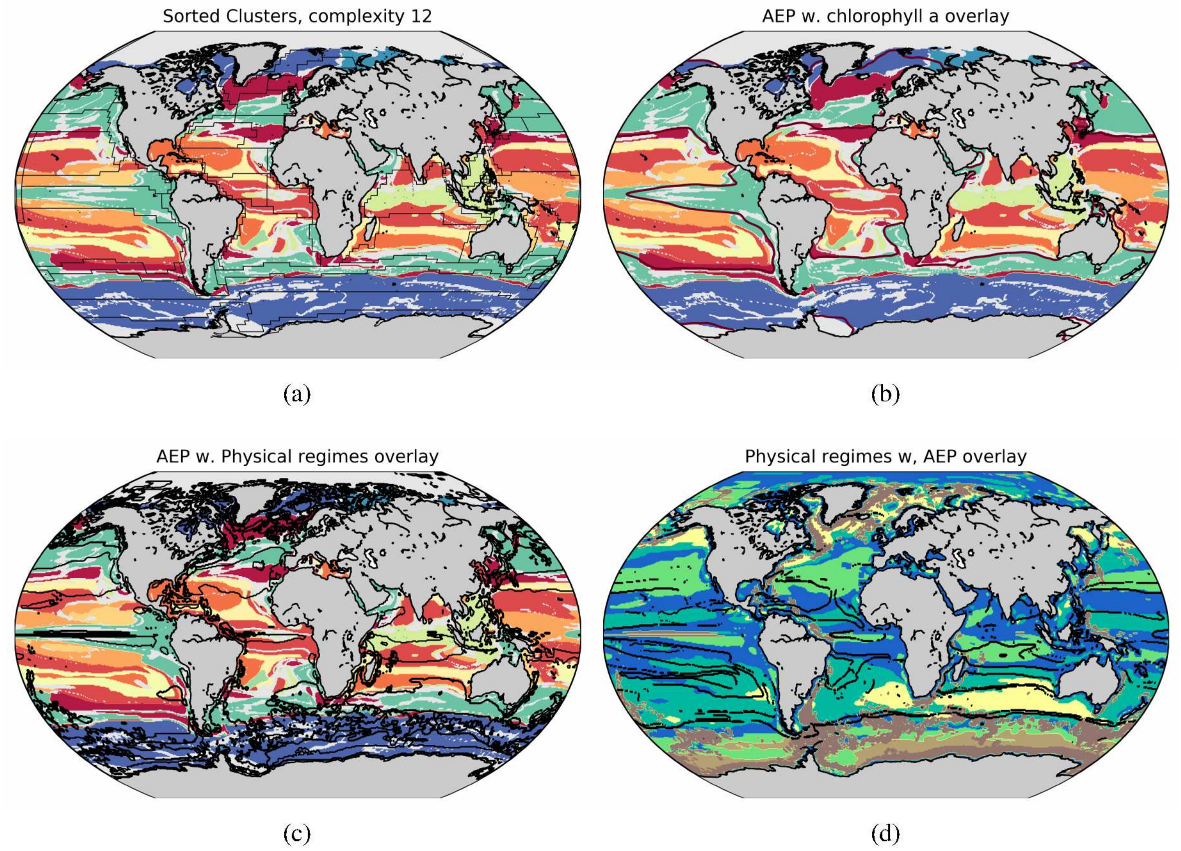

S3: Further context for the AEP of complexity 12. Comparison of the AEP complexity 12 to Longhurst (a), to the Chl-a 0.1 contour from the numerical model (b, see Fig S1), to the physical regimes in (*19*) (c), and a select number of physical regimes are overlaid (black contours) onto the AEP complexity 12 (d). Contours in c and d are overlaid as visual aids, and not all regions are shown.

ten requiring a more specialized approach. The choice of algorithm needs to be tailored to the data (e.g. DBSCAN in this study), and the results verified and validated to the extent that this is possible to avoid false positives. Starting to explore a new dataset, there is no a-priori reason to assume that the covariance ot the data is not Gaussian as a first guess, and moving to a more complicated, and potentially computationally expensive, method is first merited when simpler approaches fail. In this study, the initial analysis of the feature vector was done using methods assuming an underlying Gaussian covariance distribution; Principal component analysis (PCA) for the initial dimensionality reduction and k-means to identify clusters.

Having widespread use for dimensionality reduction (*51,52*), PCA increases interpretability and minimizes information loss. Solving an eigenvector/eigenvalue problem, PCA imposes a geometric constraint as the covariance matrix of the remaining subset of features is always diagonal, and that the data can be represented by a linear combination of the identified eigenvectors. The new uncorrelated features (variables) successively maximize variance, but the main use of PCA should be descriptive rather than inferential. For example, for spatial patterns the orthogonality constraint can give rise to spurious global structures with large amplitude even when the true pattern is known to be local.

The implication of assuming that linear combinations of the input features can capture the dominant underlying patterns, is that PCA is the optimal method for dimensionality reduction if the underlying covariance structure is Gaussian. If the input features interact non-linearly, the underlying covariance structure is likely to be non-Gaussian. These features will not be detected by the PCA. It follows that feature normalization and standardization are recommended steps.

The clustering algorithm k-means minimizes the average squared Euclidean distance from

one data point to a cluster centroid (k), where each data point belongs to the cluster with the closest mean. In our application, the types and nutrient flux data would be the dimensions/feature space that the k-means algorithm operates in. It effectively partitions the parameter space using Voronoi cells (straight lines). Relying on Euclidean distances, k-means assumes that the underlying covariance distribution associated with the clusters is Gaussian, looking for ”round” shapes. As with PCA, this assumption can mean that the algorithm fails in the presence of non-Gaussian distributions.

The clustering algorithm is used to arrive at a statistical model that can represent the process that generated the data. To assess if robust clusters have been identified in the types and nutrient flux data, the goodness-of-fit of the k-means algorithm as compared to the ”true” model that generated the data should be assessed. If successful, different numbers of parameters (k clusters) approximate the data, but the models can over, and under, fit. The optimal model in the context of k-means is the one arrived at with a number of k that closest approximates the ”true” model. If the ”true” model is known, the Kullback-Leibler divergence (cross entropy+entropy) can be used. In most cases the ”true” model is unknown, and it is common practice to assess the goodness-of-fit using information criteria. The advantage of using t-SNE comes from that the original high dimensional data is used as the ”true” model and the Kullback-Leibler divergence can in this manner probabilistically compress the high dimensional data onto lower dimensions. In this manner the topology of the data is conserved in the lower dimensional rendition.

Akaike (1971) formalised the intuition that some information is lost using a model to represent the process that generated the original data (*53*). The Akaike Information Criteria (AIC) estimates the relative amount of information lost by a given model. The AIC approximates the

”true” model by penalising an increase in candidate model complexity (for k-means increasing the number of k) using the likelyhood-function. For large volumes of data, the AIC is an asymptotically unbiased estimate of the cross-entropy risk, meaning that the model with the minimum AIC score will possess the smallest Kullback-Leibler divergence. Using this number of k gives us the statistical model that best approximates the underlying ”true” model that generated the data (*54*). The Bayesian Information Criteria (BIC) is also based on the likelihood-function, estimating a function of the posterior probability of a model being true. The BIC has a higher penalty for increasing candidate model complexity. Both the AIC and BIC are based on assumptions and asymptotic approximates, implying that they should only disagree if the AIC chooses a larger number of parameters than the BIC. For practical applications as in (*19, 22*) where k-means is used, the combination of the AIC and BIC is recommended to assess goodness-of-fit (*55*). As an example, if the BIC reaches a minimum and starts increasing, while the AIC asymptotes shortly thereafter, a parameter number between the BIC minimum and the point where the AIC asymptotes is optimal.

Both PCA and k-means minimize the mean-squared reconstruction error, and PCA is a super-sparse k-means (*56*). Applying PCA reduces the number of ”features” while preserving the variance. K-means reduces the number of data points, assigning them to the clusters, but it does **not** preserve the underlying covariance distribution of the data. This implies that k-means and PCA will agree only when the cluster centroids is sufficiently close to the PC. Note that other PCA methods exist e.g. kernel based, but are recommended only in situations where known non-linear relationships and correlations exist.

A practical example, and cautionary note, is given using the data from the Darwin model. For this data the results that the k-means algorithm produce can look reasonable (Figure S4a).

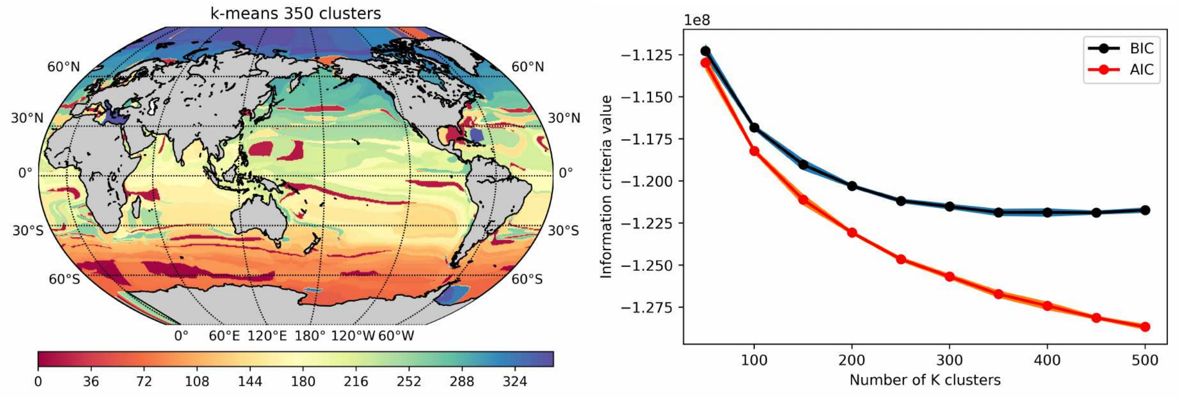

(a) Spatial projection with k=350

(b) AIC and BIC

S4: Illustration of k-means applied to the Darwin data.

Although the results visually look reasonable, the AIC and BIC test both failed, with and without PCA as a dimensionality reduction method (Figure S4b). The failure of the AIC and BIC test suggests that the underlying covariance distribution of the data is highly non-Gaussian, such that the statistical models like k-means fail to converge. This implies that the models are not able represent the underlying ”true” model, because the centroids are not able to partition the feature space so that e.g. consistent regions can be found. The suggested importance of the highly non-linear interactions in the Darwin data means that a different algorithm could be more appropriate. With the application of t-SNE, a clustering algorithm, in the Darwin model case DBSCAN, can be chosen such that emergent features of the non-linear, and non-Gaussian covariance, of the data can be captured.

REFERENCES AND NOTES

1. R. Bailey, *Ecoregions: The Ecosystem Geography of the Oceans and Continents* (Springer, 2014).

2. S. Dutkiewicz, A. E. Hickman, O. Jahn, W. W. Gregg, C. B. Mouw, M. J. Follows, Capturing optically important constituents and properties in a marine biogeochemical and ecosystem model. *Biogeosciences* **12**, 4447–4481 (2015).

3. M. D. Spalding, H. E. Fox, G. R. Allen, N. Davidson, Z. A. Ferdaña, M. Finlayson, B. S. Halpern, M. A. Jorge, A. Lombana, S. A. Lourie, K. D. Martin, E. McManus, J. Molnar, C. A. Recchia, J. Robertson, Marine Ecoregions of the World: A bioregionalization of coastal and shelf areas. *Bioscience* **57**, 573–583 (2007).

4. J. M. Omernik, G. E. Griffith, Ecoregions of the conterminous united states: Evolution of a hierarchical spatial framework. *Environ. Manag.* **54**, 1249–1266 (2014).

5. A. Longhurst, S. Sathyendranath, T. Platt, C. Caverhill, An estimate of global primary production in the ocean from satellite radiometer data. *J. Plankton Res.* **17**, 1245–1271 (1995).

6. L. Gloege, G. A. McKinley, C. B. Mouw, A. B. Ciochetto, Global evaluation of particulate organic carbon flux parameterizations and implications for atmospheric pCO_2. *Global Biogeochem. Cycles* **31**, 1192–1215 (2017).

7. J. Roff, *Marine Conservation Ecology* (Taylor & Francis, 2013).

8. R. Watson, D. Pauly, V. Christensen, R. Froese, A. Longhurst, T. Platt, S. Sathyendranath, K. Sherman, P. Celone, in *Trends in Exploitation, Protection, and Research* (Elsevier, Amsterdam, 2003), pp. 375–395.

9. IOCCG (2009). *Partition of the Ocean into Ecological Provinces: Role of Ocean-Colour Radiometry*, M. Dowell, T. Platt, Eds. (Reports of the International Ocean-Colour Coordinating Group, No. 9, IOCCG, Dartmouth, Canada, 2009).

10. T. Hattab, F. B. R. Lasram, C. Albouy, C. Sammari, M. S. Romdhane, P. Cury, F. Leprieur, F. Le Loc’h, The use of a predictive habitat model and a fuzzy logic approach for marine management and planning. *PLOS ONE* **8**, e76430 (2013).

11. M. J. Costello, P. Tsai, P. S. Wong, A. K. L. Cheung, Z. Basher, C. Chaudhary, Marine biogeographic realms and species endemicity. *Nat. Commun.* **8**, 1057 (2017).

12. M. T. Kavanaugh, B. Hales, M. Saraceno, Y. H. Spitz, A. E. White, R. M. Letelier, Hierarchical and dynamic seascapes: A quantitative framework for scaling pelagic biogeochemistry and ecology. *Prog. Oceanogr.* **120**, 291–304 (2014).

13. M. J. Oliver, A. J. Irwin, Objective global ocean biogeographic provinces. *Geophys. Res. Lett.* **35**, L15601 (2008).

14. G. Reygondeau, A. Longhurst, E. Martinez, G. Beaugrand, D. Antoine, O. Maury, Dynamic biogeochemical provinces in the global ocean. *Global Biogeochem. Cycles* **27**, 1046–1058 (2013).

15. C. B. Mouw, N. J. Hardman-Mountford, S. Alvain, A. Bracher, R. J. W. Brewin, A. Bricaud, A. M. Ciotti, E. Devred, A. Fujiwara, T. Hirata, T. Hirawake, T. S. Kostadinov, S. Roy, J. Uitz, A consumer’s guide to satellite remote sensing of multiple phytoplankton groups in the global ocean. *Front. Mar. Sci.* **4**, 41 (2017).

16. G. Lima-Mendez, K. Faust, N. Henry, J. Decelle, S. Colin, F. Carcillo, S. Chaffron, J. C. Ignacio-Espinosa, S. Roux, F. Vincent, L. Bittner, Y. Darzi, J. Wang, S. Audic, L. Berline, G. Bontempi, A. M. Cabello, L. Coppola, F. M. Cornejo-Castillo, F. d’Ovidio, L. De Meester, I. Ferrera, M.-J. Garet-Delmas, L. Guidi, E. Lara, S. Pesant, M. Royo-Llonch, G. Salazar, P. Sánchez, M. Sebastian, C. Souffreau, C. Dimier, M. Picheral, S. Searson, S. Kandels-Lewis; Tara Oceans coordinators, G. Gorsky, F. Not, H. Ogata, S. Speich, L. Stemmann, J. Weissenbach, P. Wincker, S. G. Acinas, S. Sunagawa, P. Bork, M. B. Sullivan, E. Karsenti, C. Bowler, C. de Vargas, J. Raes, Determinants of community structure in the global plankton interactome. *Science* **348**, 1262073 (2015).

17. E. T. Buitenhuis, M. Vogt, R. Moriarty, N. Bednaršek, S. C. Doney, K. Leblanc, C. Le Quéré, Y.-W. Luo, C. O’Brien, T. O’Brien, J. Peloquin, R. Schiebel, C. Swan, MAREDAT: Towards a world atlas of MARine ecosystem DATa. *Earth Syst. Sci. Data* **5**, 227–239 (2013).

18. A. Longhurst, *Ecological Geography of the Sea*, *Agricultural and Biological Sciences* (Academic Press, 1998).

19. M. Sonnewald, C. Wunsch, P. Heimbach, Unsupervised learning reveals geography of global ocean dynamical regions. *Earth Space Sci.* **6**, 784–794 (2019).

20. B. A. Ward, S. Dutkiewicz, C. M. Moore, M. J. Follows, Iron, phosphorus, and nitrogen supply ratios define the biogeography of nitrogen fixation. *Limnol. Oceanogr.* **58**, 2059–2075 2013.

21. S. Dutkiewicz, B. A. Ward, F. Monteiro, M. J. Follows, Interconnection of nitrogen fixers and iron in the pacific ocean: Theory and numerical simulations. *Global Biogeochem. Cycles* **26**, GB1012 (2012).

2. G. Maze, H. Mercier, R. Fablet, P. Tandeo, M. L. Radcenco, P. Lenca, C. Feucher, C. Le Goff, Coherent heat patterns revealed by unsupervised classification of argo temperature profiles in the North Atlantic Ocean. *Prog. Oceanogr.* **151**, 275–292 (2017).

23. D. Marmanis, M. Datcu, T. Esch, U. Stilla, Deep learning earth observation classification using ImageNet pretrained networks. *IEEE Geosci. Remote Sens. Lett.* **13**, 105–109 (2016).

24. L. van der Maaten, G. Hinton, Visualizing data using t-SNE. *J. Mach. Learn. Res.* **9**, 2579–2605 (2008).

25. S. Ghosh, K. P. Burnham, N. F. Laubscher, G. E. Dallal, L. Wilkinson, D. F. Morrison, M. W. Loyer, B. Eisenberg, S. Kullback, I. T. Jolliffe, J. S. Simonoff, Letters to the editor. *Am. Stat.* **41**, 338–341 (1987).

6. J. M. Lewis, P. M. Hull, K. Q. Weinberger, L. K. Saul, Mapping uncharted waters: Exploratory analysis, visualization, and clustering of oceanographic data, in *2008 Seventh International Conference on Machine Learning and Applications*, San Diego, CA, 11 to 13 December 2008.

27. D. Lunga, S. Prasad, M. M. Crawford, O. Ersoy, Manifold-learning-based feature extraction for classification of hyperspectral data: A review of advances in manifold learning. *IEEE Signal Process. Mag.* **31**, 55–66 (2014).

28. M. Ester, H.-P. Kriegel, J. Sander, X. Xu, in *Proceedings of the Second International Conference on Knowledge Discovery and Data Mining (KDD'96)* (AAAI Press, 1996), pp. 226–231.

29. J. R. Bray, J. Curtis, An ordination of the upland forest communities of Southern Wisconsin. *Ecol. Monogr.* **27**, 325–349 (1957).

30. M. Costanzo, B. VanderSluis, E. N. Koch, A. Baryshnikova, C. Pons, G. Tan, W. Wang, M. Usaj, J. Hanchard, S. D. Lee, V. Pelechano, E. B. Styles, M. Billmann, J. van Leeuwen, N. van Dyk, Z. Y. Lin, E. Kuzmin, J. Nelson, J. S. Piotrowski, T. Srikumar, S. Bahr, Y. Chen, R. Deshpande, C. F. Kurat, S. C. Li, Z. Li, M. M. Usaj, H. Okada, N. Pascoe, B.J. San Luis, S. Sharifpoor, E. Shuteriqi, S. W. Simpkins, J. Snider, H. G. Suresh, Y. Tan, H. Zhu, N. Malod-Dognin, V. Janjic, N. Przulj, O. G. Troyanskaya, I. Stagljar, T. Xia, Y. Ohya, A. C. Gingras, B. Raught, M. Boutros, L. M. Steinmetz, C. L. Moore, A. P. Rosebrock, A. A. Caudy, C. L. Myers, B. Andrews, C. Boone, A global genetic interaction network maps a wiring diagram of cellular function. *Science* **353**, aaf1420 (2016).

31. I. Berman-Frank, A. Quigg, Z. V. Finkel, A. J. Irwin, L. Haramaty, Nitrogen-fixation strategies and Fe requirements in cyanobacteria. *Limnol. Oceanogr.* **52**, 2260–2269 (2007).

32. S. Dutkiewicz, P. Cermeno, O. Jahn, M. J. Follows, A. E. Hickman, D. A. A. Taniguchi, B. A. Ward, Dimensions of marine phytoplankton diversity. *Biogeosciences* **17**, 609–634 (2019).

3. M. T. Kavanaugh, M. J. Church, C. O. Davis, D. M. Karl, R. M. Letelier, S. C. Doney, Aloha from the edge: Reconciling three decades of in situ Eulerian observations and geographic variability in the north pacific subtropical gyre. *Front. Mar. Sci.* **5**, 130 (2018).

34. D. M. Karl, N. R. Bates, S. Emerson, P. J. Harrison, C. Jeandel, O. Llinâs, K.-K. Liu, J.-C. Marty, A. Michaels, J. Miquel, S. Neuer, Y. Nojiri, C. S. Wong, Temporal studies of biogeochemical processes determined from ocean time-series observations during the JGOFS era, in *Ocean Biogeochemistry* (Springer, 2003).

35. L. van der Maaten, Accelerating t-SNE using tree-based algorithms. *J. Mach. Learn. Res.* **15**, 3221–3245 (2014).

36. M. Henon, C. Heiles, The applicability of the third integral of motion: Some numerical experiments. *Astron. J.* **69**, 73–79 (1964).

37. G. Forget, J.-M. Campin, P. Heimbach, C. N. Hill, R. M. Ponte, C.Wunsch, ECCO version 4: An integrated framework for non-linear inverse modeling and global ocean state estimation. *Geosci. Model Dev.* **8**, 3071–3104 (2015).

38. C. Wunsch, P. Heimbach, in *Ocean Circulation and Climate*, vol. 103 of *International Geophysics*, G. Siedler, S. M. Griffies, J. Gould, J. A. Church, Eds. (Academic Press, 2013), pp. 553–579.

39. A. Adcroft, C. Hill, J.-M. Campin, J. Marshall, P. Heimbach, *Proceedings of the ECMWF Seminar Series on Numerical Methods, Recent Developments in Numerical Methods for Atmosphere and Ocean Modelling* (ECMWF, 2004), pp. 139–149.

40. E. T. Buitenhuis, T. Hashioka, C. L. Quéré, Combined constraints on global ocean primary production using observations and models. *Global Biogeochem. Cycles* **27**, 847–858 (2013).

41. R. R. Hood, E. A. Laws, R. A. Armstrong, N. R. Bates, C. W. Brown, C. A. Carlson, F. Chai, S. C. Doney, P. G. Falkowski, R. A. Feely, M. A. M. Friedrichs, M. R. Landry, J. Keith Moore, D. M. Nelson, T. L. Richardson, B. Salihoglu, M. Schartau, D. A. Toole, J. D. Wiggert, Pelagic functional group modeling: Progress, challenges and prospects. *Deep-Sea Res. II Top. Stud. Oceanogr.* **53**, 459–512 (2006).

42. P. Tréguer, C. Bowler, B. Moriceau, S. Dutkiewicz, M. Gehlen, O. Aumont, L. Bittner, R. Dugdale, Z. Finkel, D. Iudicone, O. Jahn, L. Guidi, M. Lasbleiz, K. Leblanc, M. Levy, P. Pondaven, Influence of diatom diversity on the ocean biological carbon pump. *Nat. Geosci.* **11**, 27–37 (2018).

43. E. L. McParland, N. M. Levine, The role of differential DMSP production and community composition in predicting variability of global surface DMSP concentrations. *Limnol. Oceanogr.* **64**, 757–773 (2019).

44. A. Kuhn, S. Dutkiewicz, O. Jahn, S. Clayton, T. A. Rynearson, M. R. Mazloff, A. D. Barton, Temporal and spatial scales of correlation in marine phytoplankton communities. *J. Geophys. Res. Oceans* **124**, 9417–9438 (2019).

45. F. Pedregosa, G. Varoquaux, A. Gramfort, V. Michel, B. Thirion, O. Grisel, M. Blondel, P. Prettenhofer, R. Weiss, V. Dubourg, J. Vanderplas, A. Passos, D. Cournapeau, M. Brucher, M. Perrot, E. Duchesnay, Scikit-learn: Machine learning in Python. *J. Mach. Learn. Res.* **12**, 2825–2830 (2011).

46. T. S. Moore, J. W. Campbell, M. D. Dowell, A class-based approach to characterizing and mapping the uncertainty of the modis ocean chlorophyll product. *Remote Sens. Environ.* **113**, 2424–2430 (2009).

47. S. Clayton, S. Dutkiewicz, O. Jahn, C. Hill, P. Heimbach, M. Follows, Biogeochemical versus ecological consequences of modeled ocean physics. *Biogeosciences* **14**, 2877–2889 (2017).

48. M. Szeto, P. J. Werdell, T. S. Moore, J. W. Campbell, Are the world's oceans optically different? *J. Geophys. Res.* **116**, C00H04 (2011).

49. R. Johnson, P. G. Strutton, S. W. Wright, A. McMinn, K. M. Meiners, Three improved satellite chlorophyll algorithms for the Southern Ocean. *J. Geophys. Res. Oceans* **118**, 3694–3703 (2013).

50. C. O'Brien, J. Peloquin, M. Vogt, M. Heinle, N. Gruber, P. Ajani, H. Andruleit, J. Aristegui, L. Beaufort, M. Estrada, D. Karentz, E. Kopczynska, R. Lee, A. Poulton, T. Pritchard, C. Widdicombe, Global marine plankton functional type biomass distributions: Coccolithophores. *Earth System Science Data* **5**, 259–276 (2013).

51. I. T. Jolliffe, J. Cadima, Principal component analysis: A review and recent developments. *Philos. Trans. R. Soc. Lond. A* **374**, 20150202 (2016).

52. A. Hannachi, I. Jolliffe, D. Stephenson, Empirical orthogonal functions and related techniques in atmospheric science: A review. *Int. J. Climatol.* **27**, 1119–1152 (2007).

53. H. Akaike, Information theory and an extension of the maximum likelihood principle, in *2nd International Symposium on Information Theory*, Tsahkadsor, Armenia, USSR, 2 to 8 September 1971.

54. A.-K. Seghouane, S.-i. Amari, The AIC criterion and symmetrizing the Kullback–Leibler divergence. *IEEE Trans. Neural Netw. Learn. Syst.* **18**, 97–106 (2007).

55. J. J. Dziak, D. L. Coffman, S. T. Lanza, R. Li, L. S. Jermiin, Sensitivity and specificity of information criteria. *Brief. Bioinformatics* **21**, 553–565 (2020).

56. C. Ding, X. He, *K*-means clustering via principal component analysis, in *Proceedings of the Twenty-First International Conference on Machine Learning* (ICML'2004), Banff, Alberta, Canada, 4 to 8 July 2004.
